# Polus: a context-aware enhancement framework for DNA storage via transformer-based soft-decision decoding

**DOI:** 10.1093/bioinformatics/btag563

**Published:** 2026-07-28

**Authors:** Lulu Ding, Kun Wang, Hongmei Zhang, Shaohui Xie, Jinlong Wang, Bo Liu, Guohua Wang, Ling Liu, Zexuan Zhu

**Affiliations:** School of Artificial Intelligence, Shenzhen University, Shenzhen 518060, China; National Engineering Laboratory for Big Data System Computing, Shenzhen University, Shenzhen 518060, China; School of Artificial Intelligence, Shenzhen University, Shenzhen 518060, China; National Engineering Laboratory for Big Data System Computing, Shenzhen University, Shenzhen 518060, China; College of Computer Science and Software Engineering, Shenzhen University, Shenzhen 518060, China; College of Computer Science and Software Engineering, Shenzhen University, Shenzhen 518060, China; College of Computer Science and Software Engineering, Shenzhen University, Shenzhen 518060, China; Center for Bioinformatics, Faculty of Computing, Harbin Institute of Technology, Harbin, Heilongjiang 150001, China; Key Laboratory of Biological Bigdata, Ministry of Education, Harbin Institute of Technology, Harbin, Heilongjiang 150001, China; Center for Bioinformatics, Faculty of Computing, Harbin Institute of Technology, Harbin, Heilongjiang 150001, China; Key Laboratory of Biological Bigdata, Ministry of Education, Harbin Institute of Technology, Harbin, Heilongjiang 150001, China; Guangzhou Institute of Technology, Xidian University, Guangzhou 510700, China; School of Artificial Intelligence, Shenzhen University, Shenzhen 518060, China; National Engineering Laboratory for Big Data System Computing, Shenzhen University, Shenzhen 518060, China

## Abstract

**Motivation:**

DNA storage offers exceptional information density and archival longevity but is constrained by complex biochemical noise inherent to synthesis, storage, and sequencing. Conventional hard-decision error-correction schemes often rely on excessive redundancy to mitigate these imperfections, which significantly compromises storage efficiency and density.

**Results:**

We present Polus, a Transformer-based enhancement framework that improves digital reliability through soft-decision decoding (SDD) without requiring encoder modification. At its core is SeqFormer, a Transformer-based channel model that synergizes sequence context with quality signals to generate calibrated per-base confidence scores, effectively transforming uncertain biochemical noise into informative “soft” erasures. In *in silico* benchmarks, Polus significantly upgrades mainstream DNA storage codecs. It reduces the sequencing coverage required for DNA Fountain by 38.9%—increasing effective physical density by approximately 80%—and eliminates persistent indel-induced errors in the Yin–Yang codec. Furthermore, it enables a targeted resequencing strategy that achieves full recovery with 99.9% less overhead than uniform deepening. Moreover, a nine-metric evaluation suite was employed to provide multi-dimensional quantitative comparisons of DNA storage codecs across reliability, density, and cost. Collectively, Polus provides a reproducible framework for context-aware decoding and system design guidance for DNA storage.

**Availability and implementation:**

All source code of the Polus, including the SeqFormer implementation, codec algorithms, test data used, and the simulation pipeline is available on GitHub (https://github.com/dinglulu/Polus) and Zenodo (https://zenodo.org/communities/bioinfoszu/). A web hosted instance of Polus is available at https://polus.bioailab.net/polls/home. The SeqFormer model is also released as a standalone repository at https://github.com/dinglulu/SeqFormer and https://zenodo.org/communities/bioinfoszu/.

## 1 Introduction

The exponential growth of global data—projected to reach 400 zettabytes by 2028 (Wright 2024)—has outstripped the capacity of conventional silicon-based storage technologies. DNA storage has emerged as a compelling alternative substrate for archival storage (Ceze *et al.* 2019), offering unparalleled storage density [up to 215 petabytes per gram (Zielinski and Erlich 2017)], exceptional longevity (Grass *et al.* 2015) (millennia under appropriate conditions), and remarkable low maintenance energy (Matange *et al.* 2021). Fundamentally, DNA storage involves converting digital files into oligonucleotide sequences, synthesizing them as DNA molecules, and retrieving the data through sequencing (Meiser *et al.* 2020). However, reliable data retrieval is constrained by biochemical imperfections across the synthesis–storage–sequencing processes (Xu *et al.* 2021). DNA synthesis (e.g. phosphoramidite chemistry) introduces insertions, deletions, and substitutions due to incomplete nucleotide coupling or depurination. During storage, DNA strands may degrade (e.g. hydrolysis-driven breaks or oxidative damage causing cytosine deamination) under suboptimal conditions (Song *et al.* 2022). Sequencing adds further errors (Shendure *et al.* 2017), e.g. Illumina short-read sequencing dominates substitution error rates around 0.1%–1% (Stoler and Nekrutenko 2021), while Oxford Nanopore Technologies (ONT) long-read sequencing suffers high insertion/deletion errors (indels) with total rates of 1%–5% (Andrés-León *et al.* 2021). These heterogeneous error sources accumulate during retrieval, necessitating robust error correction frameworks to achieve DNA’s storage potential with practical reliability.

To mitigate this complex noise, DNA storage workflows typically employ two complementary forms of redundancy, i.e. physical redundancy and logical redundancy (Ding *et al.* 2024). Physical redundancy is introduced by generating multiple molecular copies via synthesizing or PCR-amplifying many copies of the same DNA strand and sequencing high coverages. Logical redundancy encodes error resilience directly into the DNA sequences through error-correcting codes (ECCs). Early proof‐of‐concept studies (Church *et al.* 2012) established simple bit-to-base mappings, yet relying on extensive physical redundancy for recovery. Subsequent efforts introduced more sophisticated schemes by integrating ECC with biochemical constraints (e.g. balanced GC content and homopolymer-run limits). Representative studies include DNA Fountain (Zielinski and Erlich 2017), concatenated Reed–Solomon (RS) schemes (Organick *et al.* 2018), and the Yin–Yang codec (YYC) (Goldman *et al.* 2013). More recent designs explicitly targeted indels by coupling synchronization with ECC, e.g. HEDGES (Press *et al.* 2020), DBGPS (Song *et al.* 2022), and LDPC–watermarking approaches (Chen *et al.* 2021). Nearly all these codecs correct errors with a hard-decision assumption, treating every base call as equally reliable. In reality, however, error rates vary across sequence contexts. For instance, long homopolymer runs or regions with secondary structure exhibit higher error propensities, and platform-specific error modes differ substantially between Illumina and ONT sequencing. Ignoring these variations compels designs to assume worst-case rates, thereby inflating redundancy requirements and limiting the efficiency of DNA storage.

A recent paradigm has been the advent of soft-decision decoding (SDD) (Ding *et al.* 2024), which replaces the binary “correct/incorrect” treatment of each base with confidence scores, demonstrating that exploiting non‐uniform base probabilities can markedly enhance decoding performance. However, the existing SDD frameworks typically assume uniform error distributions across strands and focus almost exclusively on sequencing noise, while neglecting the systematic biases from synthesis and storage. For example, in phosphoramidite synthesis the 5’-OH coupling step is markedly less efficient for guanine, leading to disproportionately high G-deletion rates (Masaki *et al.* 2022). Such context‐dependent error patterns—arising from synthesis chemistry, storage‐induced damage and platform‐specific noise—remain unexploited by existing decoders.

To address these limitations, we present Polus, an open-source enhancement framework that bridges the gap between biochemical constraints and digital reliability through context-aware SDD. At its core is a Transformer (Vaswani *et al.* 2017)-based channel model, or SeqFormer for short. SeqFormer integrates multi-copy sequence information with Phred-quality scores to generate both high-accuracy consensus sequences and calibrated per-base error probabilities. Based on the probabilistic outputs of SeqFormer, Polus provides a codec-agnostic enhancement module that significantly boosts the error-correction capacity across representative codecs—including DNA Fountain (Zielinski and Erlich 2017), YYC (Ping *et al.* 2022), and Derrick (Ding *et al.* 2024)—without requiring any modifications to their original encoders. Additionally, to facilitate more systematic cross-codec comparison, Polus incorporates a standardized nine-metric evaluation suite that quantifies key practical dimensions of DNA storage performance, including reliability, density, runtime, biochemical constraints, and cost.

Extensive benchmarking demonstrates that this enhancement framework consistently advances error identification and correction beyond existing baselines. By leveraging dual-stream multi-head self-attention within a Transformer architecture to capture long-range sequence context, SeqFormer produces calibrated per-base probabilities that enhance error enrichment by one to two orders of magnitude compared to traditional alignment-voting methods across both Illumina and ONT platforms. When deployed as an “upgrade” to existing codecs, Polus significantly reduces the sequencing redundancy required for error-free retrieval. Specifically, it lowers the coverage threshold for DNA Fountain by 38.9%—effectively increasing physical density by ∼80% (from 1.72 × 10^19^ bytes/g to 3.09 × 10^19^ bytes/g). For YYC, Polus eliminated persistent indel-induced errors that remain uncorrectable under hard-decision decoding. Furthermore, the framework’s calibrated confidence scores enable a targeted, adaptive resequencing strategy that achieves full data recovery with 99.9% less sequencing overhead than brute-force deepening. Collectively, these advances establish Polus as a quantitative and empowering framework that bridges the gap between biochemical noise and digital reliability, offering a reproducible foundation for the design of high-fidelity DNA storage systems.

## 2 Materials and methods

### 2.1 Overview of the Polus enhancement framework

**Figure 1:**
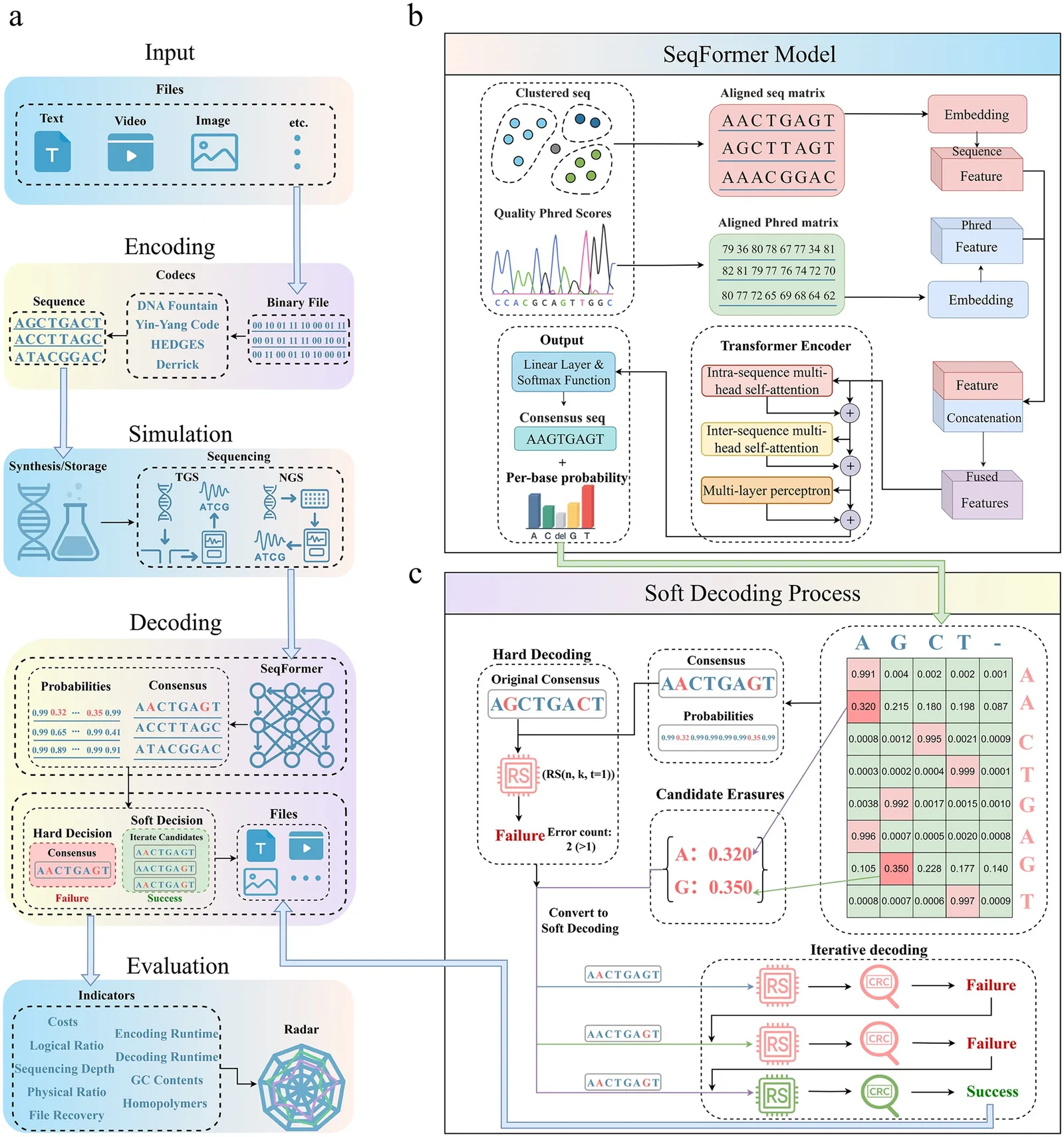
Overview of the Polus platform, the architecture of SeqFormer model and soft decoding processes. (a) The end-to-end in-silico DNA storage workflow. The pipeline integrates four modules: Encoding (converting digital files to DNA with representative codecs), Simulation (modeling synthesis, storage, and sequencing errors), Decoding, and Evaluation. (b) Schematic of the SeqFormer network. The model processes two aligned inputs: a nucleotide matrix and a corresponding Phred quality score matrix. These inputs are embedded and normalized into sequence and quality features, which are then concatenated and fused via a linear layer. (c) Schematic of soft decoding process. The decoder first takes the consensus sequence predicted by SeqFormer and performs conventional hard decoding. If decoding failure, positions with confidence scores below a predefined threshold (e.g. the highlighted A and G) are selected as candidate erasures and ranked according to their predicted error probabilities. An iterative soft-decision decoding procedure is then initiated, progressively incorporating candidate erasures in order of likelihood. The process begins by masking only the most probable error position; when this attempt fails, the erasure set is incrementally expanded. In the illustrative example, decoding succeeds only after both candidate positions are treated as erasures, at which point the recovered sequence passes CRC verification.

Polus is architected as a modular enhancement framework that establishes an end-to-end *in silico* validation and enhancement pipeline. Unlike existing storage platforms that primarily aggregate codecs (Volkel *et al.* 2023) or simulate errors (Yuan *et al.* 2022, Gimpel *et al.* 2023), Polus serves as an empowerment layer that synergizes a deep-learning-based channel engine, SeqFormer, with a codec-agnostic decoding process. The framework integrates four modular stages: namely, Encoding, Simulation, Decoding, and Evaluation into an end-to-end DNA storage pipeline (Fig. 1a). Each stage is implemented as an independent module with a standardized interface, providing a reproducible infrastructure to validate and maximize the performance of diverse DNA storage protocols without requiring any modifications to their original encoders.

For the Encoding module, digital files are converted into DNA oligonucleotide sequences using a selected codec (e.g. DNA Fountain, YYC), producing constraint-compliant sequences with addressing and parity (Supplementary Note 1, available as supplementary data at *Bioinformatics* online). During the Simulation stage, multi-source biochemical noise—synthesis errors, storage-induced damage, PCR bias and errors, and platform-specific sequencing errors—is injected to generate raw reads that mimic experimental outcomes for the encoded data. At the core of Polus is its Decoding enhancement module, which features by utilizing SeqFormer to characterize the DNA storage channel. In the process, raw sequencing reads are first processed by SeqFormer to generate both a consensus sequence and calibrated per-base error probabilities. The framework subsequently employs a dual-path mechanism: a hard-decision decoder (HDD) serves as the primary fast path, while a SDD is triggered upon HDD or cyclic redundancy check (CRC) failure. Low-confidence bases identified by the SeqFormer are flagged as erasures to enhance recovery, and the decoder iterates until it succeeds and the reconstructed file passes the CRC. Additionally, the Evaluation module computes a suite of nine quantitative metrics, encompassing file recovery rate, logical and physical density, sequencing depth, runtime, cost, GC content, and homopolymer length, to comprehensively evaluate the performance of the storage system.

### 2.2 SeqFormer model

#### 2.2.1 Model architecture

SeqFormer is a Transformer-based model designed to infer a per-base consensus sequence and a calibrated error probability for each position from a cluster of sequencing reads corresponding to one DNA oligonucleotide (Fig. 1b). From an alignment-synchronized pile of reads and their corresponding Phred quality scores, SeqFormer produces a per-oligo consensus sequence and a calibrated categorical distribution over {A, C, G, T, –} at each position (with “–” denoting a deletion). Concretely, multiple-sequence alignment information and the corresponding quality signals are jointly embedded and fused, per-read representations are aggregated across reads, and a Transformer encoder with dual-stream multi-head self-attention captures long-range sequence context before a lightweight prediction head yields per-base probabilities. The consensus sequence is then obtained by selecting, at each position, the nucleotide with the highest predicted probability, and the per-base error probability is defined as one minus the model’s probability for that chosen base.

For each oligo’s read cluster (with N reads and aligned length L), we construct two input tensors: (i) an aligned nucleotide matrix $X_{\mathrm{seq}}\in \Sigma^{N\times L}$ (Σ= {A, C, G, T, -}, where “-” denotes a gap in the multiple sequence alignment); (ii) an aligned Phred-quality matrix $X_{\mathrm{qual}}\in Q^{N\times L}$ (per-base Phred quality scores from FASTQ files, Q represents the set of ASCII characters used to encode quality values). Each branch is passed through a learned embedding layer that projects the inputs to a fixed-dimensional vector (we use an embedding dimension d=512), followed by a root-mean-square layer normalization (RMSNorm) to stabilize training across varying coverages. The per-read features from the nucleotide branch and quality branch are concatenated along the feature dimension and fused using a 1D pointwise convolution layer (kernel size: 1), yielding per-read representations $H\in R^{N\times L\times d}$. We then aggregate information by sum-pooling over reads to obtain a single fused representation $Z\in R^{L\times d}$. This aggregated sequence Z is fed into a Transformer encoder for contextual modeling. The Transformer encoder comprises 8 encoder layers, each integrating the multi-head self-attention module (8 heads) and the feed-forward module. Both submodules employ RMSNorm-based pre-normalization and residual connections. The encoder outputs a contextual embedding $\tilde{Z}\in R^{L\times d}$ that integrates long-range and cross-context dependencies that simple majority voting may miss. Finally, a linear projection followed by a softmax activation yields a probability distribution over {A, C, G, T, -} at each position. The base with the highest probability is taken as the consensus call for that position, and the softmax probability of that consensus base serves as the model’s confidence score (i.e. the estimated probability that the call is correct).

#### 2.2.2 Model training

We trained SeqFormer by minimizing the per-position cross-entropy between its predicted categorical distribution over {A, C, G, T, -} and the known designed oligo reference aligned to each read cluster. The model was implemented in PyTorch and optimized using the Adam optimizer (initial learning rate 0.0002, *β*_1_ = 0.9, *β*_2_ = 0.999). We applied a weight decay of 0.01 and dropout of 0.1 in the Transformer layers to regularize the model. Training was run for up to 5 epochs with early stopping based on validation loss to prevent overfitting. We selected the model checkpoint with the highest validation accuracy (consensus bases matching the ground truth) and best calibration (lowest deviance between predicted confidence and actual error rate). All training and inference were accelerated using an NVIDIA RTX A6000 GPU.

#### 2.2.3 Datasets and preprocessing

Because DNA sequencing error profiles differ by platform, we trained platform-specific instances of SeqFormer with identical architecture but separate weights for Illumina short reads and ONT long reads. Training used previously published DNA-storage datasets: Illumina dataset from Organick *et al.* (2018) encoding ∼200 MB and ONT dataset from Ding *et al.* (2024) encoding ∼5 MB. Each dataset was split 3:1 into training and test sets. Accession codes and download links are provided in Table S1, available as supplementary data at *Bioinformatics* online. For each dataset, raw FASTQ files were demultiplexed and adapter-trimmed, then clustered by oligonucleotide address to form per-oligo read piles. Each pile was aligned using BSAlign to generate a multiple sequence alignment, with gaps represented as “-”; the corresponding per-base Phred quality scores were retained and mapped to the alignment coordinates.

#### 2.2.4 Model selection

SeqFormer is distributed as a single executable with a command-line option to select the pretrained model: ONT-trained is invoked with “SeqFormer --tgs”, and Illumina-trained with “SeqFormer --ngs”. Unless specified otherwise, we used frozen weights without additional fine-tuning.

### 2.3 SDD implementation

After SeqFormer produces a consensus sequence and an error probability for each base of an oligo, we then feed this information into the decoding algorithms of Fountain, YYC, or Derrick as appropriate, modifying each decoder to accept “soft” inputs. Across all three pipelines, soft information is ultimately consumed at an RS layer (Fig. 1c).

#### 2.3.1 Principle

An RS code over n symbols (with k data symbols) can correct up to t=[(n-k)/2] errors (or 2t erasures), and more generally can successfully decode if the condition 2e+s≤(n-k) is met (where s is the number of erasures supplied to the decoder and e is the number of residual errors). We leveraged SeqFormer’s base-wise confidence to identify likely error positions in the consensus. Specifically, we define a confidence threshold θ (by default θ=0.1) on SeqFormer’s predicted error probabilities. Any base with $P_{\mathrm{error}}>\theta$ is marked as a candidate erasure. These candidates are ranked by their error probability to form an erasure set E. We then iteratively select subsets of E for RS decoding, gradually pruning the least likely erasures until a valid codeword is obtained. Successful decodings are verified against a CRC32 checksum, which ensures that undetected RS collisions cannot propagate as false positives.

#### 2.3.2 Threshold and calibration

SeqFormer’s confidences are strongly polarized in practice (correct bases typically>0.9). A sweep of θ ∈ [0, 0.2] shows a broad optimum around θ=0.1, which maximizes full-file recovery by balancing false erasures against missed errors. We set θ=0.1 as default.

#### 2.3.3 Codec-specific mapping

In DNA Fountain, base-level confidences within each droplet are aggregated to byte level: bytes exceeding θ are erased before inner RS decoding, and only RS-passed droplets are forwarded to LT solving. In YYC, the outer RS code spans strands; per-strand symbols inherit from their protected segments, and soft erasures allow the parity code to focus capacity on genuinely ambiguous symbols. In Derrick, fixed-length payload sequences form a matrix; RS is applied across sequences (columns). For each column, symbol probability comes from the aligned base window of that column; erasures are flagged column-wise and decoded vertically.

### 2.4 Standardized evaluation suite

To compare performance across schemes comprehensively, Polus implements nine standardized metrics:

#### 2.4.1 File recovery rate

Calculated via bit recovery rate (BRR)–the fraction of bits that are correct in the final reconstructed file (after decoding) relative to the original input–and block recovery rate (LRR)–the percentage of codeword blocks that are decoded without any errors. In our context, a 100% BRR or LRR means the file is perfectly decoded.

#### 2.4.2 Logical density

The number of information bits stored per nucleotide synthesized. Let $B_{\mathrm{payload}}$ be the number of information bits in the input file(s), and let $N_{nt}$ be the total number of synthesized nucleotides (sum over all oligos, including addressing and ECC). Logical density is


(1) $$D_{\mathrm{logical}}=\frac{B_{\mathrm{payload}}}{N_{nt}}(\mathrm{bits}/nt).\;$$

#### 2.4.3 Physical density

The amount of data stored per unit mass of DNA, typically expressed in bytes per gram. First, under an ideal dry-DNA assumption, the number of nucleotides per gram is ${\;N}_{A}/M_{nt}$, where $N_{A}=6.02\times 10^{23}\;{\mathrm{mol}}^{-1}$ is the SI-defining Avogadro constant and $M_{nt}$ is the average molar mass per nucleotide of ssDNA (we use the standard approximation $M_{nt}=330\;g\cdot {\mathrm{mol}}^{-1}\cdot {nt}^{-1}$). Thus, the ideal physical density is computed by


(2) $$D_{\mathrm{phys},\mathrm{ideal}}=\frac{D_{\mathrm{logical}}}{8}\cdot \frac{N_{A}}{M_{nt}}(\mathrm{bytes}/g).\;$$

Then, actual physical density for ssDNA. In practice, reliable readout requires multiple copy numbers per oligo, C, which we take as the minimal average coverage that yielded error-free file recovery in our pipeline (from our *in-silico* experiments). Under the assumptions of the storage of 100 molecules per oligo sequence (Church *et al.* 2012), no synthesis/long-term loss (effective yield $f_{\mathrm{yield}}=1$), the actual density reduces to $D_{\mathrm{phys}}=D_{\mathrm{phys},\mathrm{ideal}}/\left(C\times 100\right)$. More details about the computation were added in the Supplementary Note 4, available as supplementary data at *Bioinformatics* online.

#### 2.4.4 Sequencing depth requirement

The minimum average sequencing coverage per oligo required to consistently recover the data without errors. We determine this by running simulations with decreasing read depths until decoding fails.

#### 2.4.5 Encoding runtime

We measured wall-clock times of the encoder implementations on an Intel Xeon Platinum 8352 V processor to compare their efficiency.

#### 2.4.6 Decoding runtime

We measured wall-clock times of the decoder implementations on an Intel Xeon Platinum 8352 V processor to compare their efficiency. The clustering, SeqFormer inference (for Polus), and error correction decoding time were separately recorded.

#### 2.4.7 Costs

An estimate of the monetary cost to store and retrieve the data using each scheme, factoring in DNA synthesis cost (per base, given the logical redundancy) and sequencing cost (per read, given the depth needed). We used current synthesis prices (∼$0.10 per base) and sequencing prices ($1000 per ∼1 billion reads for Illumina, ∼$30 per GB for Nanopore MinION) as a basis (Shen *et al.* 2025).

#### 2.4.8 GC contents

The average (and distribution of) GC fraction in the encoded oligos. All our schemes enforced roughly 45%–55% GC content, but we included this to ensure none produce extreme GC outliers that could impede PCR or sequencing.

#### 2.4.9 Maximum homopolymer length

The longest run of identical nucleotides in any encoded oligo. This is a measure of biochemical constraint adherence, as long homopolymers (e.g. >5 bases) can cause higher sequencing errors.

## 3 Results

In this section, we first validate SeqFormer’s architecture, consensus accuracy, and calibration performance, then demonstrate Polus’s consistent SDD gains with DNA Fountain, YYC, and Derrick codecs, and finally compare these codecs using the nine-metric evaluation suite.

### 3.1 Effects of SeqFormer

#### 3.1.1 Consensus accuracy

**Figure 2:**
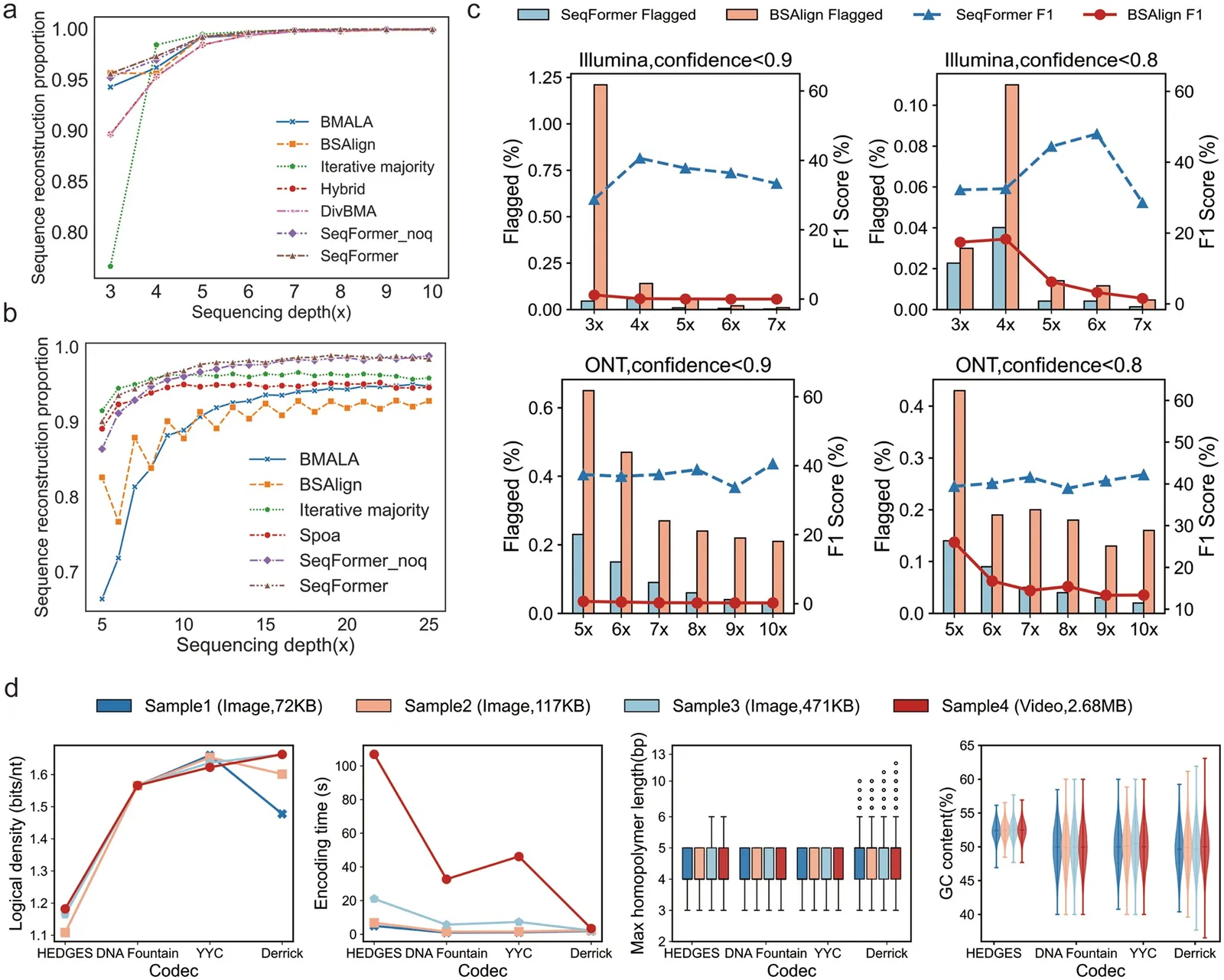
Consensus reconstruction, confidence-based error enrichment, and codec encoding assessment. (a, b) Sequence reconstruction proportion across sequencing depths for Illumina-like (a) and ONT-like (b) datasets. SeqFormer was compared with representative alignment-based methods, including Iterative majority, DivBMA (only Illumina), Hybrid (only Illumina), BSAlign, Spoa (only ONT), and BMALA where applicable. SeqFormer-noq denotes an ablated SeqFormer model in which Phred quality-score input was removed while multi-read sequence-context input was retained. (c) Confidence-based error enrichment under Illumina-like and ONT-like conditions using confidence thresholds of 0.9 and 0.8. Bars indicate the fraction of bases flagged as low-confidence candidate erasures, and lines indicate the F1-score for error detection. Compared with BSAlign-derived confidence estimates, SeqFormer maintained higher error-detection performance while flagging an orders-of-magnitude smaller fraction of bases. (d) Encoding characteristics of four DNA storage codecs (HEDGES, DNA Fountain, YYC, and Derrick) using four file samples ranging from 72 KB to 2.68 MB. Subpanels show logical density (bits/nt), encoding time (s), the distribution of maximum homopolymer length, and GC content distribution of the encoded sequences.

SeqFormer enables high-accuracy consensus calling with lower sequencing depth than previous methods (Fig. 2a and b). On the Illumina-sequenced DNA oligonucleotides from Organick *et al.* (Organick *et al.* 2018) (encoding ∼200 MB data), SeqFormer reconstructed ∼95.7% of oligonucleotides error-free at only 3× coverage, slightly surpassing BSAlign (Shao and Ruan 2024) and BMALA (Ceze *et al.* 2018) and clearly outperforming Iterative Majority, DivBMA, and Hybrid (Sabary *et al.* 2024) methods. By 5× coverage, all methods exceeded 98% accuracy, with SeqFormer and Iterative Majority both reaching ∼99.4%. Although Iterative Majority can slightly outperform SeqFormer at higher coverage (≥4×), its runtime grows steeply with depth, making SeqFormer far more efficient at practical coverages. Similarly, on the ONT-sequenced DNA oligonucleotides from Ding *et al.* (2024) (encoding ∼5 MB data), SeqFormer remained competitive at very low depth and became the top performer beyond ∼9× coverage, exceeding BMALA’s accuracy by 5%–20% within comparable runtime (<100 s).

#### 3.1.2 Ablation analysis of SeqFormer inputs

To further dissect the contributions of Phred quality scores and sequence-context modeling, we introduced an ablated model, SeqFormer-noq, in which the Phred quality-score branch was removed while the multi-read sequence-context input and Transformer architecture were retained. On the Illumina dataset (Fig. 2a), SeqFormer-noq achieved reconstruction performance comparable to that of the full SeqFormer model across 3×–10× coverage, likely because the relatively low error rate of Illumina reads allows multi-read sequence evidence alone to provide strong consensus information. On the ONT dataset (Fig. 2b), where errors are more frequent and indel-rich, SeqFormer-noq consistently outperformed conventional heuristic consensus baselines across most sequencing depths, indicating that the Transformer architecture can capture informative context-dependent error patterns from multi-read sequence evidence. Nevertheless, the full SeqFormer model further improved upon SeqFormer-noq, particularly when sequencing coverage was limited (5×–10×), demonstrating that Phred quality scores provide complementary information when integrated with sequence context and inter-read evidence. Together, these results support the design of SeqFormer as a dual-input channel model rather than a Phred-score-only confidence estimator.

#### 3.1.3 Calibration of base-wise probabilities

The probabilistic outputs of SeqFormer are well-calibrated, with output confidence scores closely reflecting actual error probabilities (Fig. S1 and S2, available as supplementary data at *Bioinformatics* online). On the Illumina dataset across 3× to 7× coverage, bases in SeqFormer’s high-confidence bin (0.9–1.0) had empirical error rate dropping from 2.27 × 10^−4^ at 3× coverage to undetectable at 7× coverage. In contrast, the baseline BSAlign became increasingly overconfident, i.e. its high-confidence error rate increased from 1.81 × 10^−3^ to 4.45 × 10^−3^ over the same range, presumably because simple vote-based heuristics inflate confidence with read count even when systematic errors persist (Nakamura *et al.* 2011, Ross *et al.* 2013). Consequently, in the high-confidence regime, SeqFormer was ≥8-fold better calibrated at 3× coverage and ≥300-fold at 6 × coverage than BSAlign, with zero high-confidence errors at 7 × coverage. A similar trend was observed on ONT datasets, i.e. the high-confidence error rate of SeqFormer fell by an order of magnitude as coverage increased, whereas that of BSAlign rose—implying a SeqFormer calibration advantage of ≥43-fold at 5× coverage rising to ≥560-fold at 10× coverage (Fig. S2, available as supplementary data at *Bioinformatics* online).

#### 3.1.4 Error enrichment

The confidence scores of SeqFormer allow most true errors to be isolated in a small candidate subset for downstream correction. Across different platforms and depths, SeqFormer delivers consistently higher F1 scores that take both precision and recall into account than the alignment-based BSAlign (Fig. 2c;  Fig. S3, available as supplementary data at *Bioinformatics* online). Using a pragmatic threshold of 0.9 (flagging bases with confidence < 0.9 as candidate erasures), Polus peaks at F1 score with 40.6% under 4× coverage on the Illumina dataset while flagging 0.06% of bases, and 10× coverage with only 0.03% of bases flagged on the ONT dataset. Tightening the threshold to 0.8 further improved SeqFormer’s precision without sacrificing recall, yielding F1 up to 42.2% while keeping the flagged set below 0.14% of positions on the ONT dataset. These results indicate that SeqFormer provides far stronger error enrichment with orders-of-magnitude smaller flagged bases across platforms and coverages, yielding more informative erasure hints for downstream SDD.

**Figure 3:**
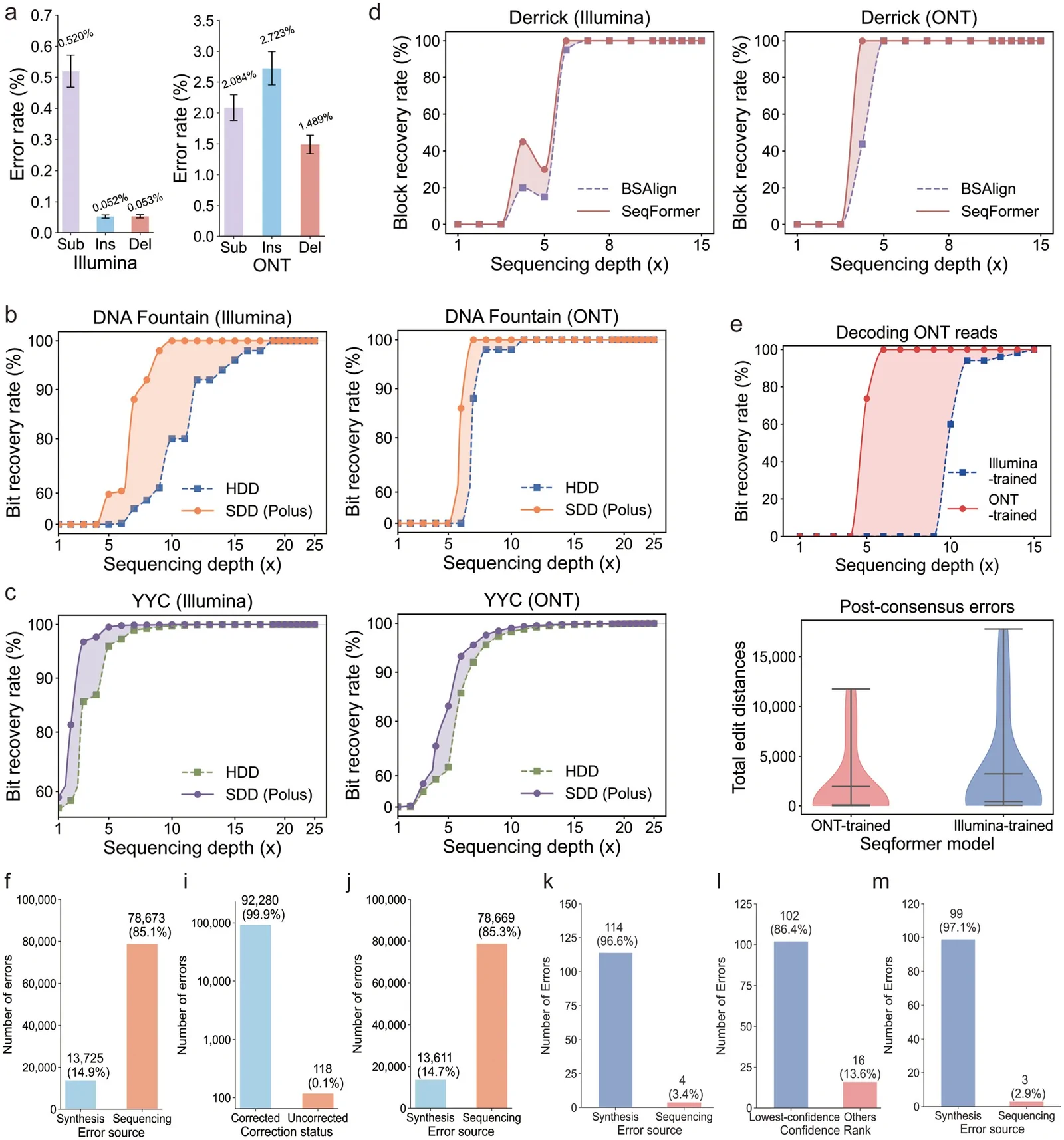
Soft-decision decoding gains and source attribution analysis under simulated platform-specific error profiles. (a) Error-type distributions generated by the Polus simulation module for Illumina- and ONT-like platforms. (b, c) Bit recovery rates across sequencing depths for DNA Fountain (b) and YYC (c) under Illumina-like and ONT-like error profiles, comparing hard-decision decoding (HDD, dashed lines) with Polus-enhanced soft-decision decoding (SDD, solid lines). (d) Block recovery rates of the Derrick pipeline using BSAlign or SeqFormer for consensus generation and confidence scores under Illumina-like and ONT-like error profiles. (e) Effect of model specificity on ONT decoding, showing post-consensus edit-distance distributions and bit recovery rates for ONT reads decoded with ONT-trained or Illumina-trained SeqFormer models. (f–m) Source-attribution analysis showing that SeqFormer corrects most sequencing- and synthesis-derived errors during consensus generation, while residual errors—predominantly synthesis-derived—are enriched among low-confidence positions and can be flagged as erasures for downstream soft-decision decoding.

### 3.2 Encoding module performance

The encoding stage of Polus revealed codec-specific performance tradeoffs on sample files (Fig. 2d). For example, the logical density (information bits per nucleotide, i.e. bits/nt) of YYC declines as file size grows because longer indices are needed to address more oligonucleotides, making YYC more efficient for smaller datasets. In contrast, the logical density of Derrick improves with larger files as its fixed matrix padding overhead becomes proportionally smaller, favoring application on larger files. HEDGES and DNA Fountain show nearly size-invariant density. In terms of speed, Derrick is the fastest mainly thanks to the implementation in *C* language. All codecs produced ∼50% GC content on average. Fountain, YYC, and HEDGES enforce homopolymer runs ≤ 6 nt, whereas Derrick relies on randomness and occasionally has slightly longer runs. By reporting these metrics, Polus enables informed codec selection based on application-specific priorities such as density, constraint stringency, and speed.

### 3.3 Multi-stage error simulation

Polus integrates a modular, *in silico* pipeline that simulates errors arising from synthesis, storage, amplification, and sequencing (Supplementary Note 2, available as supplementary data at *Bioinformatics* online), enabling real-world DNA storage error landscapes. Unlike prior platforms restricted to Illumina-like substitution errors (Gimpel *et al.* 2023), Polus extends simulation to both short-read and nanopore long-read profiles by integrating the simulators dt4dds and Badread (Wick 2019), respectively. The simulated error statistics match the known platform differences, i.e. under Illumina-like conditions, substitutions dominate (5.2 × 10^−3^ per base on average) with indels an order of magnitude rarer (mostly single-base events), whereas ONT-like simulations show higher overall error rates (∼5.3%) with indels prevalent (Fig. 3a; Figs S4 and S5, available as supplementary data at *Bioinformatics* online).

**Figure 4:**
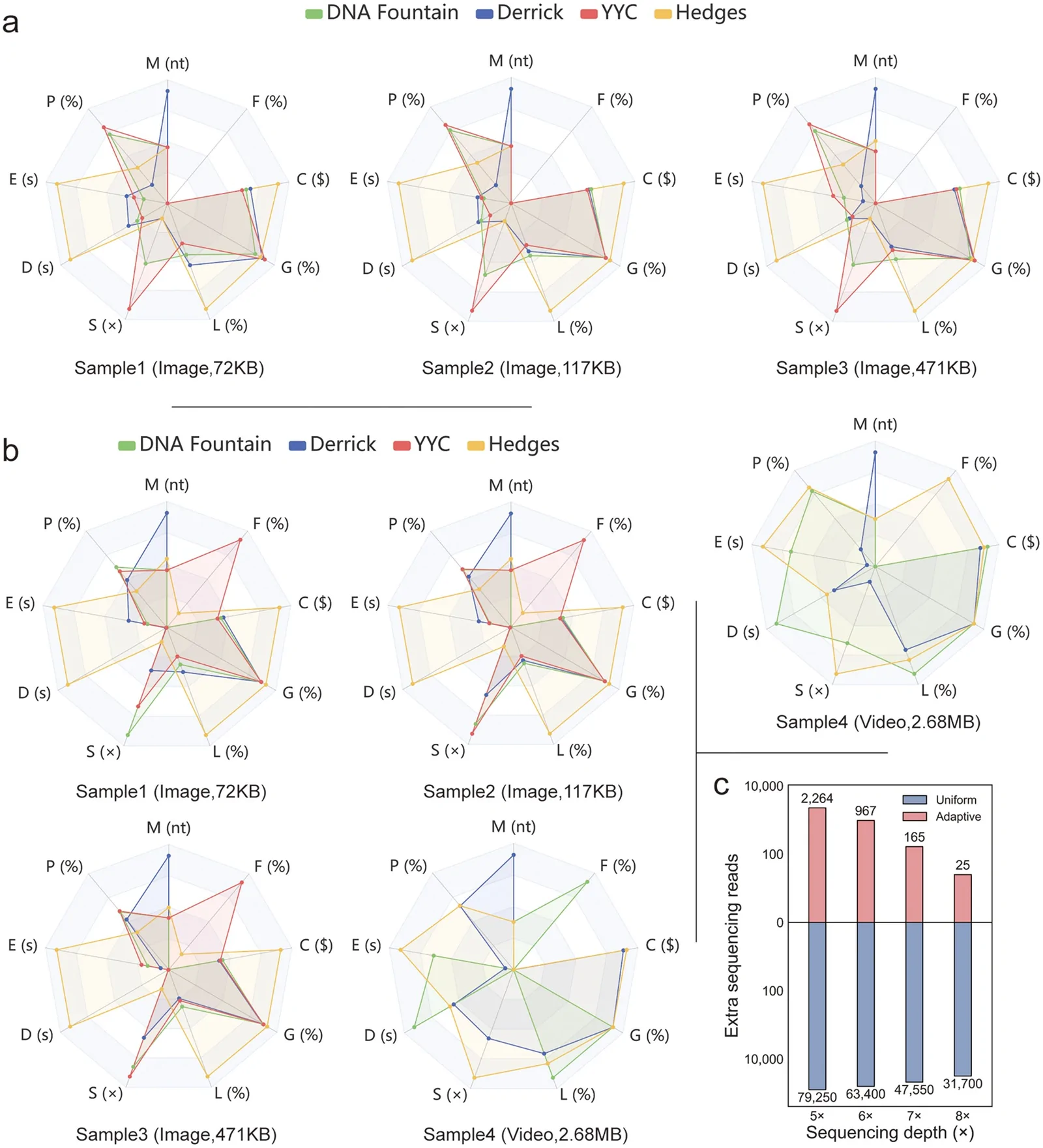
Multi-dimensional performance evaluation and adaptive resequencing efficiency. (a, b) Radar charts summarizing the tradeoffs of four codecs (DNA Fountain, YYC, HEDGES, Derrick) under (a) Illumina and (b) ONT error profiles. Axes represent nine standardized metrics: File recovery rate (F), Cost (C), Sequencing depth (S), Encoding time (E), Decoding time (D), GC deviation (G), and Max homopolymer length (M), Physical density shortfall (P, defined as the percentage of the theoretical physical storage density (455 EB/g) not achieved by a given scheme; smaller values indicate densities closer to the theoretical limit.), and Logical density shortfall (L, defined as the percentage of the theoretical maximum (2 bits/nt) not achieved). (c) Comparison of additional sequencing reads required to achieve full recovery using Polus’s adaptive strategy versus a uniform deepening baseline.

### 3.4 Decoding module and soft decoding gains

In the Decoding stage, Polus uses the error probability outputs of SeqFormer to implement SDD across different codecs, significantly extending their error-correction capability. Rather than treating each base as equally reliable, Polus leverages the per-base posterior probabilities of SeqFormer to mark low-confidence loci as erasures. Since RS codes correct erasures more efficiently than unmarked errors, this approach increases effective ECC headroom without changing the encoding. Consistently, integrating SeqFormer with existing codecs reduced the sequencing depth required for perfect recovery, lowered associated costs, and enhanced the achievable physical storage density in all tested schemes (Table 1).

**Table 1:** Overall decoding performance improvements with Polus’ SDD.^a^

Codec	Decoder	Depth (×)	Physical density (bytes/g)	File recovery rate	Cost ($)	Runtime (s)	Logical density
**Illumina (108 KB encoded file)**							
DNA Fountain	**SDD (Polus)**	**11**	**3.09 × 10^19^**	**100%**	**97.95**	**0.86**	1.357
	HDD (baseline)	18	1.72 × 10^19^	100%	99.21	0.89	
YYC	**SDD (Polus)**	**16**	**1.94 × 10^19^**	**100%**	**98.58**	0.07	1.361
	HDD (baseline)	—	—	99.98%	—	0.07	
Derrick	**SDD (Polus)**	**6**	**6.06 × 10^19^**	**100%**	**1120.68**	27.96	**1.593**
	SDD (baseline)	7	5.19 × 10^19^	100%	1122.5	**10.4**	
**ONT (108 KB encoded file)**							
DNA Fountain	**SDD (Polus)**	**7**	**4.28 × 10^19^**	**100%**	**517.71**	1.02	1.315
	HDD (baseline)	11	2.73 × 10^19^	100%	520.13	**0.94**	
YYC	**SDD (Polus)**	**24**	**1.35 × 10^19^**	**100%**	**488.95**	0.58	1.42
	HDD (baseline)	—	—	99.98%	—	0.58	
Derrick	**SDD (Polus)**	**5**	**9.09 × 10^19^**	**100%**	**1115.01**	35.85	**1.593**
	SDD (baseline)	6	7.27 × 10^19^	98.75%	1116.32	**17.18**	

a On *in-silico* Illumina and ONT datasets, soft-decision decoding (SDD; Polus) reduced the required sequencing depth and increased physical density relative to hard-decision decoders for DNA Fountain, YYC, and BSAlign-guided SDD for Derrick, while preserving perfect file recovery and comparable cost and runtime. Boldface denotes the better result within each platform–codec pair; “—” indicates HDD failed to achieve perfect recovery even with additional sequencing depths.

#### 3.4.1 DNA Fountain with SDD

For DNA Fountain codec, SeqFormer-guided SDD markedly lowered the coverage needed for perfect data retrieval under both Illumina and ONT error profiles (Fig. 3b). With an inner RS (36, 35) code on Illumina-like data, Polus achieved 100% recovery at 11× coverage, whereas the hard-decision decoder left ∼2% of bits unrecovered at ∼17× coverage and required 18× coverage to fully succeed. Under nanopore-like errors (RS (36, 34)), Polus reached full recovery at 7× coverage versus 11× coverage for hard decoding. At 6× coverage, hard decoding failed entirely while Polus can still reconstruct more than 80% of the files. Thus, context-aware erasures substantially reduced the minimum coverage and improved recovery at lower depths.

#### 3.4.2 YYC with SDD

For YYC codec, SeqFormer-guided SDD eliminated residual indel errors that the original hard-decoder could not correct (Fig. 3c). Under Illumina-like conditions, two oligonucleotides carrying terminal 2 bp insertions prevented complete recovery even beyond 25× coverage with hard decoding. SeqFormer identified these anomalous insertions with low confidence and marked them as erasures, allowing the outer RS decoder to repair both strands and achieve full retrieval at 20× coverage. Across moderate depths (e.g. 6× coverage), Polus consistently improved recovery rate (to >99% versus ∼97% with hard decoding). Similarly, with ONT-like noise, hard decoding plateaued just below 100% recovery at 25× coverage due to uncorrectable indels, whereas SDD reached 100% recovery at 24× coverage by erasing those errors.

#### 3.4.3 Derrick with Polus

In the RS (255,223) Derrick scheme, SeqFormer-based decoding reduced the required coverage for error-free recovery (Fig. 3d) and improved physical density, while maintaining comparable runtime (Table 1), in contrast to the original BSAlign-guided pipeline. Across all three codecs, these results illustrated that probabilistic soft decoding universally improved reliability, lowered redundancy, and cut down sequencing requirements for error-free recovery.

### 3.5 Adaptive decoding performance

In practical application, well-calibrated confidence estimates enable an efficient adaptive sequencing strategy to minimize laboratory effort. We simulated this with a DNA Fountain dataset (317 oligonucleotides protected by RS (36, 35)). For each starting coverage (5× to 8×), 50 independent resampling were generated. As a baseline, uniform deepening to 11× coverage was required to achieve 100% recovery with the Polus SDD pipeline. In the adaptive setting (Supplementary Note 3, available as supplementary data at *Bioinformatics* online), we began at the selected starting coverage, identified decode-failed oligonucleotides, and iteratively requested oligo-specific additional reads. After each increment we recomputed SeqFormer’s estimated consensus accuracy and attempted decoding only when this estimate exceeded 0.998. If decoding fails, another increment was performed. Each oligonucleotide cluster underwent at most five adaptive iterations.

This adaptive strategy consistently achieved 100% file recovery while using orders-of-magnitude fewer additional reads than uniform deepening (Fig. 4c;  Table S1, available as supplementary data at *Bioinformatics* online). Across 5× to 8× coverage, the adaptive procedure required 40 to 3,891 additional reads in total, corresponding to 99.92% to 95.91% fewer than the uniform-deepening baseline at the same starting point. Thus, computational guidance via SeqFormer can substitute for brute-force sequencing, preserving data integrity with minimal sequencing overhead.

### 3.6 Platform-specific SeqFormer improves decoding

Tailoring SeqFormer to the sequencing platform further boosted decoding performance (Fig. 3d). We compared two models—one trained on Illumina reads and one on ONT reads—for decoding an ONT test dataset. The ONT-trained model produced ∼40% fewer post-consensus errors on average and achieved full data recovery at 7× coverage, whereas the Illumina-trained model required 15× coverage (a 53.3% higher depth). The results demonstrate that platform-matched training is critical for maintaining calibration and maximizing decoding gains. In practice, we recommend adopting SeqFormer trained on the sequencing technology of interest and fine-tuning on similar error profiles when possible, to fully leverage probabilistic decoding’s advantages.

### 3.7 Source attribution of SeqFormer-corrected and residual errors

To clarify how SeqFormer interacts with different noise sources, we established an error-source attribution analysis that tracked errors from raw readouts through SeqFormer consensus generation and confidence-based flagging (Fig. 3f–m). Before correction, the raw readout error pool consisted predominantly of sequencing-induced errors (85.1%), with a smaller fraction attributed to synthesis-derived errors (14.9%) (Fig. 3f). After SeqFormer consensus generation, 92,280 errors were corrected, corresponding to 99.9% of all observed errors (Fig. 3i). These corrected errors retained a similar source composition, including 78,669 sequencing-induced errors (85.3%) and 13,611 synthesis-derived errors (14.7%) (Fig. 3j), indicating that SeqFormer corrects errors from both sources when they are resolvable from multi-read sequence and quality evidence. Among the 118 residual errors that remained after correction, synthesis-derived errors were strongly enriched, accounting for 114 positions (96.6%) (Fig. 3k). Notably, 102 of these residual errors (86.4%) were ranked among the three lowest-confidence positions predicted by SeqFormer (Fig. 3l), and 99 of these low-confidence residual errors (97.1%) were synthesis-derived (Fig. 3m). These results indicate that SeqFormer not only corrects the vast majority of sequencing- and synthesis-derived errors during consensus generation, but also assigns low confidence to many unresolved positions, enabling them to be treated as erasures for downstream soft-decision decoding.

### 3.8 Evaluation metrics module

Polus’s evaluation module benchmarked each codec across nine metrics capturing retrieval fidelity, storage density, cost, and biochemical constraints. The metrics are defined in detailed in Section 2. This multi-dimensional comparison revealed tradeoffs that would be obscured by any single-metric assessments. Under Illumina-like conditions, YYC achieved the highest logical density on small files and lowest synthesis cost, but required higher sequencing coverage (lower effective physical density) (Fig. 4a). In contrast, Derrick provided better coverage efficiency on small files and dominated large files (≥471 KB) with the highest logical density and consequently the greatest physical density. Under ONT-like error conditions, similar trends held. Notably, DNA Fountain failed to recover the largest dataset (∼2.6 MB) due to indel-induced frame shifts that its hard-decision RS decoding could not correct (Fig. 4b). HEDGES attained the highest physical density (owing to a minimal 2× coverage requirement) but suffered the lowest logical density and about double the overall cost of other schemes.

Two quantitative trends emerged from cross-platform analysis. First, total costs correlated negatively with logical density, because synthesis dominated expenditure at an approximate 1000:1 cost ratio relative to sequencing (Xu *et al.* 2024). Second, when sequencing depth was incorporated into physical density estimation, the effective physical density was determined primarily by the combined effects of information density and sequencing depth. Because logical density (upper bound ≈2 bits per nt) was less impactful than differences in depth, our results indicated that effective physical density is inversely correlated with sequencing depth. This further implies that, for a given encoding scheme, enhancing the decoder’s error-correction capability not only reduces total cost but also substantially increases physical density.

## 4 Discussion

By learning calibrated per-base error probabilities from multimodal read evidence, Polus converts uncertain base calls into erasures that can subsequently be resolved by downstream Reed–Solomon (RS) decoding. Across representative DNA storage pipelines, including DNA Fountain (Zielinski and Erlich 2017), YYC (Ping *et al.* 2022), and Derrick (Ding *et al.* 2024), this confidence-aware decoding strategy consistently reduced the sequencing depth required for lossless recovery. To mitigate false-positive decoding outcomes arising from short-length RS codes, CRC32 validation was incorporated to identify cases in which RS decoding returns a decoded valid codeword but the reconstructed payload remains incorrect. Under Illumina-like simulation conditions, Polus reduced the sequencing coverage required for complete recovery in the DNA Fountain workflow from 18× to 10× and resolved persistent indel-associated failures observed in YYC decoding pipelines (BER 0.03%→0) under hard-decision decoding.

FrameD (Volkel *et al.* 2023) and DeSP (Yuan *et al.* 2022) provide valuable infrastructure for simulation, benchmarking, and design-space exploration of DNA storage pipelines. These frameworks mainly support the evaluation of codec performance under different synthesis, sequencing, and error-model conditions. Polus is complementary to these efforts. Rather than serving as another benchmarking platform, Polus is designed as a downstream decoding enhancement framework that improves recovery robustness through confidence-aware soft-decision inference. In addition, the modular structure of Polus enables SeqFormer-based confidence estimation and soft-decision decoding to be incorporated into both established and emerging DNA storage systems without altering their original encoding architectures.

These decoding gains are primarily enabled by SeqFormer, which integrates sequence context and quality-related channel features within a Transformer-based architecture to generate calibrated base-level confidence estimates. Low-confidence loci are subsequently treated as erasures during RS decoding, thereby extending the effective correction capability without increasing redundancy. Importantly, certain errors, particularly indel-dominated events, persist even at increased sequencing depth under conventional hard-decision decoding schemes; soft-decision inputs, however, enable the outer code to reconcile these events, which explains the observed reduction in coverage thresholds for zero-loss recovery. Beyond DNA storage decoding, SeqFormer may also have broader utility for consensus generation and related bioinformatics applications.

Recent studies have explored probabilistic and machine-learning-assisted decoding strategies for DNA storage, including the integration of neural-network-derived confidence information with downstream decoding algorithms (Zheng *et al.* 2024). These advances collectively highlight the value of confidence-aware decoding under noisy sequencing conditions. In contrast to approaches coupled to specific coding schemes (Jeong *et al.* 2024) or sequencing pipelines (Chandak 2020, Volkel *et al.* 2024), Polus is designed as a codec-compatible soft-decision enhancement layer that can be readily integrated into diverse existing DNA storage systems.

An important future direction will be extending SeqFormer to expanded molecular alphabets, particularly composite letters, which suffer from elevated error rates and exhibit strong letter-specific transition biases. By exploiting cross-read contextual correlations, SeqFormer could more accurately calibrate transition tendencies among highly confusable letters, thereby improving inference fidelity. In addition, integrating SeqFormer’s context-aware posterior probabilities with prior transition models (transition libraries) could further enable prior-guided re-ranking of candidate letters, effectively reconciling probabilistic predictions with the molecular error profiles arising from synthesis and sequencing. Taken together, these strategies outline a principled route to reduce sequencing depth requirements and enhance robustness in composite letter-based DNA storage systems. More broadly, coupling confidence-aware decoding with automated laboratory workflows may enable adaptive decisions between resequencing, rewriting, and decoding in real time. Together, these developments may contribute to more reliable and cost-efficient DNA storage systems, while preserving codec diversity.

## Supplementary Material

btag563_Supplementary_Data

## Data Availability

All the datasets used for training and evaluation were obtained from public databases. The Illumina sequencing datasets from Organick *et al.*(Organick *et al.* 2018) and the Nanopore sequencing datasets from Ding *et al.*(Ding *et al.* 2024) used to train SeqFormer can be obtained via their supplementary materials; we provide processed versions (read alignments and per-base data) in our repository to facilitate model training. The accession numbers and data links are listed in Table S2, available as supplementary data at *Bioinformatics* online. All simulated data generated during this study (including encoded oligo libraries, simulated FASTQ reads, and decoder outputs) can be regenerated using the code and parameter settings we have released. The sample image and video files for Polus pipeline tests, decoding performance and evaluation performance are provided at https://github.com/dinglulu/Polus/tree/main/test_data
